# Trajectories of Metabolic Risk Factors and Biochemical Markers prior to the Onset of Cardiovascular Disease – The Doetinchem Cohort Study

**DOI:** 10.1371/journal.pone.0155978

**Published:** 2016-05-20

**Authors:** Gerben Hulsegge, Annemieke M. W. Spijkerman, Yvonne T. van der Schouw, Stephan J. L. Bakker, Ron T. Gansevoort, Henriette A. Smit, W. M. Monique Verschuren

**Affiliations:** 1 Centre for Nutrition, Prevention and Health Services, National Institute of Public Health and the Environment, Bilthoven, The Netherlands; 2 Julius Center for Health Sciences and Primary Care, University Medical Center Utrecht, Utrecht, The Netherlands; 3 Department of Internal Medicine, Division of Nephrology, University Medical Center Groningen and University of Groningen, Groningen, The Netherlands; University of the Balearic Islands, SPAIN

## Abstract

Risk factors often develop at young age and are maintained over time, but it is not fully understood how risk factors develop over time preceding cardiovascular disease (CVD). Our objective was to examine how levels and trajectories of metabolic risk factors and biochemical markers prior to diagnosis differ between people with and without CVD over a period of up to 15–20 years. A total of 449 incident non-fatal and fatal CVD cases and 1,347 age- and sex-matched controls were identified in a prospective cohort between 1993 and 2011. Metabolic risk factors and biochemical markers were measured at five-year intervals prior to diagnosis. Trajectories of metabolic risk factors and biochemical markers were analysed using random coefficient analyses. Although not always statistically significant, participants with CVD had slightly more unfavourable levels for most metabolic risk factors and biochemical markers 15–20 years before diagnosis than controls. Subsequent trajectories until diagnosis were similar in participants with incident CVD and controls for body mass index, diastolic blood pressure, total cholesterol, HDL cholesterol, random glucose, triglycerides, gamma glutamyltransferase, C-reactive protein and uric acid. Trajectories were more unfavourable in participants with CVD than controls for systolic blood pressure, waist circumference and estimated glomerular filtration rate (p≤0.05). For example, among participants with CVD, systolic blood pressure increased on average by 9 mmHg over the 18-year period preceding diagnosis, whereas the increase among controls was 4 mmHg. In conclusion, unfavourable levels of metabolic risk factors and biochemical markers are present long before CVD, which indicates that the risk of CVD is already partly determined in young adulthood. This underscores the need for early prevention to reduce the burden of CVD.

## Introduction

It is unclear whether cardiovascular disease (CVD) is preceded by gradual accumulation of adverse levels of risk factors starting at young age, by relatively sudden deterioration in risk factors shortly before disease onset, or a combination of both. Although it has been well-established that adverse levels of risk factors often develop early in life and are maintained over time [1–6], it is not fully understood as to how they progress to CVD. For a better understanding of the natural history of CVD, it is necessary to know how metabolic risk factors and biochemical markers develop during the decades prior to the onset of CVD. The comparison of long-term trajectories of metabolic risk factors and biochemical markers between those who do and those who do not develop CVD will provide insight into the timing and the extent of pathophysiological changes before the occurrence of CVD, which may, therefore, give an indication as to the optimal timing of preventive actions.

Long-term trajectories of metabolic risk factors and biochemical markers preceding CVD have hardly been explored but the few available studies suggest differences between those with and those without CVD long before diagnosis. For example, at the age of 17, males in the Israel Defence Forces who later developed a stenosis of more than 50% in at least one coronary artery had similar BMI levels to the men without coronary stenosis; however, subsequent increases in BMI up to diagnosis at the age of 25–45 years were larger in men who developed the stenosis than among other young men.[7] In the Whitehall II study, British civil servants with CVD had higher levels of C-reactive protein (CRP) 14 years prior to diagnosis of fatal CVD than those individuals without CVD [8]. This difference remained similar until the occurrence of CVD. Insight into trajectories of other important CVD risk factors, such as blood pressure [9], lipids [10, 11], liver fat accumulation [12] and kidney function [13] may lead to a better understanding of the long-term physiological changes preceding CVD. Therefore, we examined how levels and trajectories of several metabolic risk factors and biochemical markers prior to diagnosis among initially healthy men and women differed between those who later developed CVD and those who did not, over a period of up to 15–20 years.

## Materials and Methods

### Population

The Doetinchem Cohort Study is an ongoing population-based longitudinal study of men and women aged 20–59 years from Doetinchem, a town in the Netherlands. Men and women were invited to undergo a clinical examination from 1987–1991 (wave 1: N = 7,768, participation rate: 62%), 1993–1997 (wave 2: N = 6,117), 1998–2002 (wave 3: N = 4,918), 2003–2007 (wave 4: N = 4,520) and 2008–2012 (wave 5: N = 4,018). Response rates were 75% or higher in waves 2–5. Details are described elsewhere.[14] We excluded 2,250 participants from the current analyses based on the following exclusion criteria: participation in only one wave (N = 1,378); missing follow up information on CVD or no informed consent for linkage with Statistics Netherlands or the Dutch Hospital Discharge Registry (n = 416); prevalent CVD at baseline or wave 2 based on hospital discharge data and self-reporting (N = 184); missing data on biochemical markers in all waves due to absence of informed consent to use blood samples for future research (N = 90); and non-participation in the wave prior to diagnosis of CVD (N = 182). This led to a population of 2,517 men and 3,001 women. Pregnant women were excluded for the wave in which they were pregnant. All participants gave written informed consent for each wave and the study was approved according to the guidelines of the Helsinki Declaration by the Medical Ethics Committee of the University Medical Center Utrecht.

### Measurements

Weight, height, waist circumference, diastolic and systolic blood pressure were measured and blood samples were taken according to standard protocols [14]. Body weight was measured to the nearest 0.1 kg on calibrated scales and 1 kg was subtracted to adjust for clothing; height was measured to the nearest 0.5 cm. BMI was calculated as weight divided by height squared (kg/m^2^). Waist circumference was measured twice to the nearest 0.5 cm, at the level midway between the lowest rib and the iliac crest at the end of expiration, with participants in standing position. The mean of two measurements was used for analysis. Plasma glucose levels were determined in random non-fasting venous blood samples using the hexokinase method [15]. Diastolic and systolic blood pressure levels were measured twice after 2 minutes of rest and the average of these two measurements was used in the analyses. Participants were measured in sitting position with a random zero sphygmomanometer (Hawksley and Sons, Lancing, UK) in waves one through three. In waves four and five a Speidel Keller meter (Welch Allyn, Skaneateles Falls, NY, USA) was used. Mean diastolic and systolic blood pressure levels measured during wave four were unexpectedly higher compared to the blood pressure levels in the previous and following waves. No clear cause could be identified, therefore, we statistically corrected blood pressure values of wave 4, as described extensively elsewhere [16]. Total cholesterol and HDL cholesterol were measured until 1998 in non-fasting EDTA-plasma and from 1998 onwards in serum, using standardised enzymatic methods. In 2013–2014, standardised enzymatic methods (Roche/Hitachi Modular P analyser, Mannheim, Germany) were used to retrospectively determine additional biochemical markers from waves 2–5 in non-fasting plasma samples that had been stored at -20 degree Celsius until June 1995 and at -80 degree Celsius from July 1995 onwards. Gamma-glutamyltransferase (GGT), uric acid, triglycerides (GPO-PAP assay) and alanine aminotransferase (ALT) (kinetic UV assay) were measured with a colorimetric method. ALT measurements were excluded until June 1995 (N = 2,495) because prior to that blood plasma was stored at -20 degree Celsius, a temperature at which ALT has poor stability [15]. ALT (N≤2), and GGT (N≤29) values greater than three times the upper normal reference were recoded as missing for that wave since this may indicate liver problems [17]. High sensitivity CRP was measured with the principle of particle-enhanced immunological agglutination (Tina-quant CRP) and cystatin C measurement was based on a particle enhanced-turbidimetric immunoassay using reagents from Gentian (Gentian, Moss, Norway). CRP values above 10 mg/L were recoded as missing for that wave because this may have indicated an acute-phase response to infection, for example, or physical injury rather than chronic subclinical inflammation (N≤80) [18]. Creatinine was measured with a Creatinine Plus assay (IDMS traceable). Estimated glomerular filtration rate (eGFR) was estimated with the Chronic Disease Epidemiology Collaboration (CKD-EPI) equation using a combination of cystatin C and creatinine [19]. Data on educational attainment, smoking status and use of anti-hypertensive and cholesterol-lowering medication were obtained by questionnaire.

### Cardiovascular disease

Non-fatal and fatal cardiovascular events that occurred after the second examination wave were ascertained until January 1, 2011. Cause of death was ascertained through linkage with Statistics Netherlands, and morbidity data were obtained through probabilistic linkage with the Dutch Hospital Discharge Registry [20]. We defined fatal CVD cases (where CVD was the primary or secondary cause of death) and non-fatal CVD cases according to ICD-9 codes 410–414, 415.1, 427.5, 428, 430–438, 440–442, 443.9, 444, 798.1, 798.2, 798.9 [21] and corresponding ICD-10 codes [22].

### Selection of controls

For each incident CVD case (N = 449), three controls were randomly selected from the same study wave and matched on age (±3 years) and sex using incidence density sampling, which is the preferred method for a nested case-control design and was recently also proposed for retrospective, longitudinal analyses [23, 24]. This led to a 1,347 controls. We matched to control as much as possible for differences in metabolic risk factors and biochemical markers between those with and those without CVD caused by differences in age and length of follow-up.

### Data analysis

The two-sample t-test and chi-square test were used to assess differences in baseline variables between individuals with CVD and controls.

From the date of diagnosis, participants were followed back in time for 5–24 years (Table 1), i.e. participants diagnosed between waves 2–3, 3–4, 4–5 and after wave 5 could be followed back in time for 6–11, 11–16, 16–21 or 21–24 years respectively. BMI, blood pressure, total cholesterol and HDL cholesterol were followed back in time for a maximum of 24 years, and other metabolic risk factors and biochemical markers were followed back in time for a maximum of 18 years, as those factors were not measured during the first examination wave.

**Table 1:** Number of incident CVD cases at each wave and the corresponding follow-up time of metabolic risk factors and biochemical markers.

Moment of diagnosis	Number of incident CVD cases	Number of years prior to diagnosis that measurements of BMI, DBP, SBP, TC and HDLc were available	Number of years prior to diagnosis that measurements of glucose, WC, TG, ALT, GGT, CRP, UA, eGFR were available	Age at diagnosis Mean ± standard deviation
Between waves 2–3	120	6–11	0–5	55.4 ± 8.2
Between waves 3–4	128	11–16	5–10	59.3 ± 8.6
Between waves 4–5	163	16–21	10–15	64.0 ± 9.1
After wave 5	38	21–24	15–18	63.5 ± 8.8

Abbreviations: BMI, body mass index; CVD, cardiovascular disease; DBP, diastolic blood pressure; SBP, systolic blood pressure; TC, total cholesterol; HDLc, high-density lipoprotein cholesterol; WC, waist circumference; TG, triglycerides; ALT, alanine aminotransferase; GGT, gamma glutamyltransferase; CRP, C-reactive protein; UA, uric acid; eGFR, estimated glomerular filtration rate

Note: For example, cases diagnosed after wave 5 had measurements of BMI, DBP, SBP, TC and HDLc for up to 21–24 years and measurements of other risk factors and biochemical markers for up to 15–18 years prior to diagnosis. Cases diagnosed between waves 3–4 had measurements of BMI, DBP, SBP, TC and HDLc for up to 11–16 years and measurements of other risk factors and biochemical markers for up to 5–10 years prior to diagnosis.

Trajectories were constructed by random coefficient analyses adjusted for sex, age (linear, plus quadratic and cubic age-terms if it statistically significantly improved model fit), examination wave and time prior to event as a linear function for each metabolic risk factor and biochemical marker (dependent variable) separately. For participants with incident CVD, the exact time of each examination prior to diagnosis was calculated by subtracting the examination date from the date of diagnosis of CVD. Matched controls were assigned the same follow-up time as their respective cases. Age was centred at 60 years, which was approximately the mean age at wave 5, and examination wave was centred at wave 4 to fit trajectories for a hypothetical population of 60-year-olds in 2002–2007 (T_0_). We centred the examination wave at wave 4 and not wave 5 for optimal power since there were only few incident CVD cases after wave 5 (N = 38). Trajectories of diastolic and systolic blood pressure were also adjusted for anti-hypertensive medications, and trajectories of total cholesterol, HDL cholesterol and triglycerides were adjusted for cholesterol-lowering medications. We log-transformed triglycerides, ALT, GGT and CRP and reported back-transformed geometric means since these biochemical markers did not have a normal distribution.

Non-linearity was investigated by fitting first-order fractional polynomials of time [25]. The following transformations of time before event were fitted: linear, quadratic, cubic, square root, logarithmic, and the inverse of linear, quadratic and square root. A non-linear function of time replaced the linear function of time when the model fit was statistically significantly better based on the likelihood ratio test (P<0.05). A second polynomial was included in the model when inclusion further improved the model fit.

We tested differences between individuals with CVD and controls in 1) levels of metabolic risk factors and biochemical markers 18 years before CVD, 2) subsequent trajectories until diagnosis and 3) levels at diagnosis. Participants with incident CVD and controls were contrasted using the contrast statement in SAS to test differences in levels of metabolic risk factors and biochemical markers at diagnosis and 18 years prior to diagnosis, the maximum follow-up time of most metabolic risk factors and biochemical markers. Differences in trajectories of metabolic risk factors and biochemical markers between participants with incident CVD and controls were statistically tested (P<0.10) using an interaction term or interaction terms for the interaction(s) between CVD status and the time function(s) (i.e. time before event). A three-way interaction between age, CVD status and the time function(s) tested whether differences in trajectories between individuals with CVD and controls differed significantly by age (P<0.10).

We had limited power to stratify the primary analyses by specific CVD endpoint (coronary heart disease, stroke). To get an indication of potential differences in trajectories between subtypes of CVD, we modelled trajectories of BMI, diastolic and systolic blood pressure, total cholesterol and HDL cholesterol separately for coronary heart disease and stroke as a sensitivity analysis. These were chosen because they are major risk factors of CVD, and the full follow-up period was available for these risk factors. Trajectories were additionally adjusted for BMI and centred at 25 kg/m^2^ in sensitivity analyses to investigate whether differences in trajectories between participants with incident CVD and controls could be explained by BMI, a key driver of the other metabolic risk factors and biochemical markers [26–29]. All analyses were performed using SAS 9.3 software.

## Results

In the total study population, 449 participants developed CVD (290 men; 159 women): 281 coronary heart disease events, 117 strokes and 51 other cardiovascular events occurred. For participants with incident CVD and controls, blood pressure, total cholesterol, HDL cholesterol and BMI (that were measured from the first wave onwards) were followed back in time for an average of 2.9 waves and 14.9 years (range: 6.0–23.8) while the other risk factors and biochemical markers (that were measured from the second wave onwards) were followed back in time for an average of 2.2 waves and 11.1 years (range: 5.0–17.8). Participants diagnosed with CVD later during follow-up were older (Table 1). At baseline, the average age was 45.4 (range: 20.1–59.8). Participants with incident CVD were more likely to have had a lower level of education and to have been a smoker than controls (Table 2). At each wave, levels of the risk factors and biochemical markers were slightly more unfavourable in participants with incident CVD than in controls (S1 Table).

**Table 2:** Baseline characteristics of participants who developed cardiovascular disease and those who did not.

	Cardiovascular disease (N = 449)	No cardiovascular disease (N = 1,347)	P-value for difference
**Demographics**			
Age (years), mean (SD)	45.4 (8.7)	45.5 (8.7)	0.57
Women (%)	159 (35%)	477 (35%)	1.00
Low educational level (%)*	304 (68%)	804 (60%)	0.03
**Smoking status**			
Currently smoking (%)	198 (44%)	372 (28%)	<0.01
Ex-smoker (%)	123 (27%)	475 (35%)	<0.01
**Medication**			
Anti-hypertensive medication (%)	29 (6%)	56 (4%)	0.06
Cholesterol-lowering medication (%)	0 (0%)	1 (0%)	0.30

* Intermediate secondary education or less.

### Differences in initial levels of metabolic risk factors and biochemical markers between participants with CVD and controls

**Fig 1:**
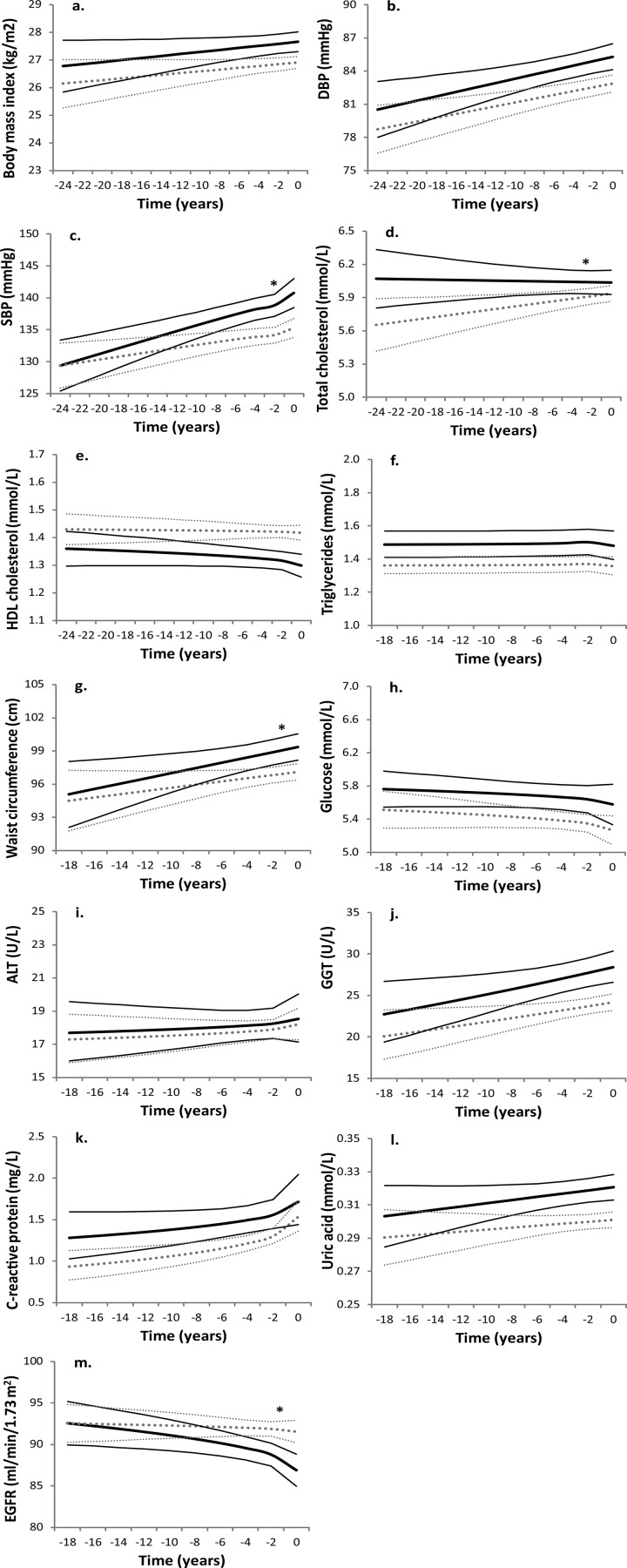
**Trajectories of body mass index (a), DBP (b), SBP (c), total cholesterol (d), HDL cholesterol (e), triglycerides (f), waist circumference (g), random glucose (h), ALT (i), GGT (j), C-reactive protein (k), Uric acid (l), and eGFR (m) of those participants with incident cardiovascular disease (solid black lines) and controls (dashed grey lines) for a hypothetical population of 60 year olds at diagnosis.** Abbreviations: DBP, diastolic blood pressure; SBP, systolic blood pressure; ALT, alanine aminotransferase; GGT, gamma glutamyltransferase; eGFR, estimated glomerular filtration rate. The thin black lines represent the 95% confidence intervals of mean levels of metabolic risk factors and biochemical markers for individuals with CVD. The thin dashed grey lines represent the 95% confidence intervals of mean levels of metabolic risk factors and biochemical markers for controls. Geometric means are shown for triglycerides, alanine aminotransferase, gamma glutamyltransferase and C-reactive protein. An asterisk (*) indicates a statistically significant difference in trajectory between cases and controls (P<0.10).

With the exception of systolic blood pressure, waist circumference, ALT and eGFR, participants with incident CVD had, on average, slightly more unfavourable levels of metabolic risk factors and biochemical markers than controls at 18 years before diagnosis in age-adjusted analyses (Fig 1,Table 3 and S2 Table). This difference was only statistically significantly for HDL cholesterol, triglycerides and random glucose (P<0.05).

**Table 3:** Mean levels of metabolic risk factors and biochemical markers 18 years prior to diagnosis of cardiovascular disease and at diagnosis, and percentage change during that period, separately for cases and controls.

	Mean level at T_-18_*			Mean level at diagnosis*			Percentage change between T_-18_ and diagnosis		
	Cases	Controls	P-value for difference	Cases	Controls	P-value for difference	Cases	Controls	P-value for difference †
Body mass index (kg/m^2^)	27.0	26.3	NS	27.7	26.9	<0.01	2%	2%	NS
Diastolic blood pressure (mmHg)	82	80	NS	85	83	<0.01	4%	4%	NS
Systolic blood pressure (mmHg)	132	131	NS	141	135	<0.01	7%	3%	<0.01
Total cholesterol (mmol/L)	6.1	5.7	NS	6.0	5.9	NS	0%	4%	<0.05
HDL cholesterol (mmol/L)	1.35	1.43	<0.05	1.30	1.42	<0.01	-3%	-1%	NS
Triglycerides (mmol/L)	1.5	1.4	<0.01	1.5	1.4	<0.01	0%	0%	NS
Glucose (mmol/L)	5.8	5.5	<0.01	5.6	5.3	<0.05	-3%	-4%	NS
Waist circumference (cm)	95	95	NS	99	97	<0.01	4%	2%	0.05
ALT (U/L)	18	17	NS	19	18	NS	5%	5%	NS
GGT (U/L)	22	20	NS	28	24	<0.01	25%	20%	NS
C-reactive protein (mg/L)	1.3	0.9	NS	1.7	1.5	NS	34%	64%	NS
Uric acid (mmol/L)	0.30	0.29	NS	0.32	0.30	<0.01	6%	4%	NS
eGFR (ml/min/1.73 m^2^)	93	93	NS	87	92	<0.01	-6%	-1%	<0.01

Abbreviations: ALT, alanine aminotransferase; GGT, gamma glutamyltransferase; eGFR, estimated glomerular filtration rate; NS: not significant.

*Age-adjusted mean levels were estimated using random coefficient analyses.

†Difference in trajectory was statistically tested with interaction term(s) between time prior to diagnosis and cardiovascular disease status.

### Differences in trajectories of metabolic risk factors and biochemical markers between participants with CVD and controls

The differences observed between participants with incident CVD and controls 15–20 years before diagnosis remained stable over time for most metabolic risk factors and biochemical markers (P≥0.10) (Fig 1 and Table 3). During the 18 years preceding diagnosis, in both participants with incident CVD and controls, increasing levels were seen for mean BMI (2%), diastolic blood pressure (4%), random glucose (3–4%), ALT (5%), GGT (20–25%), CRP (34–64%) and uric acid (4–6%); decreasing levels for HDL cholesterol (-3 to -4%); and stable levels for triglycerides. In individuals with CVD, this resulted in statistically significantly more unfavourable levels of BMI, diastolic blood pressure, HDL cholesterol, triglycerides, random glucose, GGT and uric acid at diagnosis compared to controls (P<0.01).

Levels of ALT, CRP and total cholesterol were not statistically significantly different in participants with CVD and controls 15–20 years before diagnosis and at the end of follow-up/at diagnosis (P≥0.05). Trajectories of ALT and CRP were similar in participants with CVD and controls (P≥0.10), while the trajectory of total cholesterol was statistically significantly less unfavourable in individuals with incident CVD than controls (P<0.05). The difference in change in total cholesterol between participants with CVD and controls became 40% smaller after adjustment for cholesterol-lowering medication but remained statistically significantly different (P<0.05).

More unfavourable trajectories were observed in participants with CVD than in controls for systolic blood pressure (P<0.01), waist circumference (P = 0.05) and eGFR (P<0.01) (Fig 1 and Table 3). The similar levels of these risk factors in participants with CVD and controls 15–20 years before CVD slowly turned into more unfavourable levels in those with CVD at diagnosis (P<0.01). In men and women with incident CVD the mean systolic blood pressure increased from 132 to 141 mmHg (7%) in the 18 years preceding diagnosis, while systolic blood pressure increased from 131 to 135 mmHg (3%) in controls. During the same period, waist circumference increased from 95 to 99 cm (4%) and eGFR decreased from 93 to 87 ml/min/1.73m^2^ (-6%) in participants with incident CVD, while changes were 2–6 times smaller in controls.

There were no statistically significant differences by age in the comparison of trajectories of metabolic risk factors and biochemical markers between people with CVD and controls (P≥0.10 for three-way interactions), with the exception of HDL cholesterol (P = 0.07 for three-interaction) and ALT (P = 0.02 for three-way interaction).

### Sensitivity analyses

**Fig 2:**
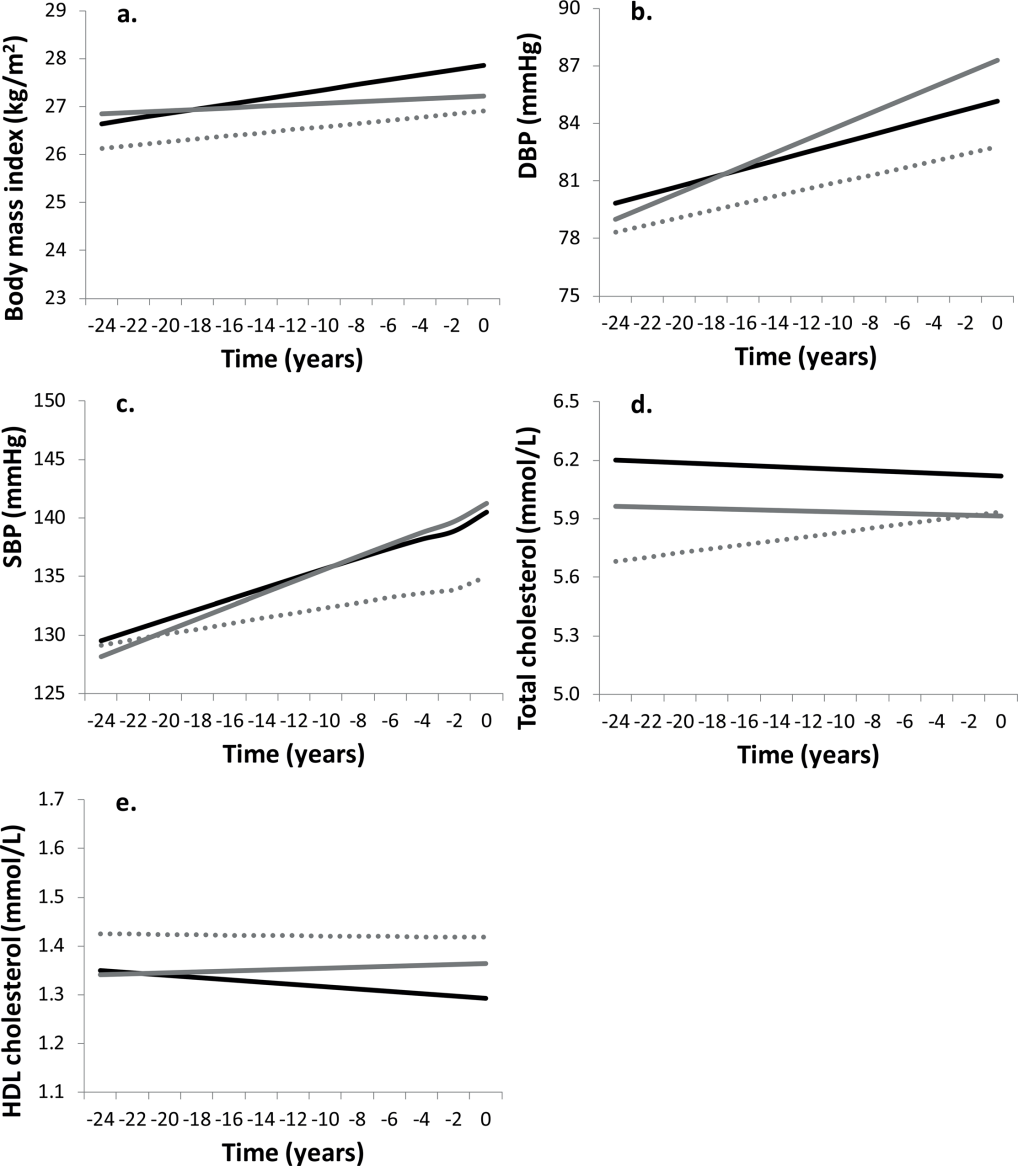
Trajectories of body mass index (a), diastolic blood pressure (b), systolic blood pressure (c), total cholesterol (d) and HDL cholesterol (e) of those participants with incident coronary heart disease (solid black lines), incident stroke (solid grey lines) and controls (dashed grey lines) for a hypothetical population of 60 year olds at diagnosis.

Participants with coronary heart disease and stroke had no statistically significantly different levels or trajectories of BMI, diastolic and blood pressure, total cholesterol and HDL cholesterol during the 15–20 years prior to diagnosis (Fig 2). Other sensitivity analyses showed that adjustment for BMI only slightly attenuated differences in metabolic risk factors and biochemical markers between participants with incident CVD and controls (S1 Fig).

## Discussion

Long before (up to 15–20 years prior to) the diagnosis of CVD, people with incident CVD had, although not always statistically significant, slightly more unfavourable metabolic risk factor and biochemical marker levels than controls. Subsequent trajectories in BMI, diastolic blood pressure, HDL cholesterol, random glucose, triglycerides, GGT, ALT, CRP and uric acid were similar in people with incident CVD and controls until diagnosis. For systolic blood pressure, waist circumference and eGFR, similar levels were seen at 15–20 years before diagnosis, but more unfavourable subsequent trajectories were observed for participants with incident CVD compared to controls.

Differences in all metabolic risk factors and biochemical markers were small in our population but are in line with results from the INTERHEART study that also revealed small differences in mean levels of BMI (0.3 kg/m2) and HDL cholesterol (0.03 mmol/L) between individuals with myocardial infarction and controls [30, 31]. Although we did not observe a statistically significant difference in CRP between individuals with CVD and controls, the mean difference in CRP (0.2-0-4 mg/L) over the 18-year period is also in accordance with findings from the Whitehall II study. They showed a constant difference in mean CRP of 0.3–0.4 mg/L between people with incident fatal CVD and controls during the 14 years prior to diagnosis [8]. Extending the findings of the Whitehall II study to other metabolic risk factors and biochemical markers, our results suggest that differences in most other metabolic risk factors and biochemical markers were already present long before diagnosis, and, in general, subsequent trajectories were similar between individuals with CVD and controls up to the occurrence of CVD. These differences were not explained by differences in BMI, and, in general, did not differ by age at diagnosis. These findings underscore the fact that CVD is caused by a long-term multifactorial disease process in which adverse effects of elevated levels of multiple risk factors are already present at young age and slowly accumulate over time, rather than being the result of rapidly increasing levels of risk factors in the years preceding diagnosis.

We observed a larger increase in total cholesterol in controls than in participants with CVD. This may be the result of a larger increase in the use of statins in individuals with CVD than controls. Indeed, in participants with CVD use of cholesterol-lowering medication was 50% higher than in controls. Although additional adjustment for cholesterol-lowering medication attenuated the difference in trajectory of total cholesterol between cases and controls by 40%, it may not fully account for this increase in statin use.

During the 15–20 years preceding CVD, the course of only two of the metabolic risk factors and one of the biochemical markers was different in individuals with incident CVD than in controls: increases in systolic blood pressure, waist circumference and eGFR were larger in participants with incident CVD than in controls. This suggests that unfavourable changes in these three risk factors in the 15–20 years prior to diagnosis might be important in the development of CVD. Independently from BMI, waist circumference is associated with systolic blood pressure and GFR [32, 33], which suggests that the unfavourable trajectory in waist circumference partially drives the unfavourable trajectories of blood pressure and eGFR. Blood pressure and GFR are also highly related, and each may adversely affect the other [34, 35]. The trajectory of eGFR may, therefore, resemble the trajectory of systolic blood pressure, and vice versa. In addition, unfavourable changes in either GFR or systolic blood pressure may also exacerbate the effect of the other. Although the unfavourable trajectories of systolic blood pressure, waist circumference and eGFR in people with incident CVD are biological plausible, the possibility of chance findings cannot be ruled out.

The more unfavourable trajectory in waist circumference but not in BMI in participants with incident CVD in comparison to controls suggests that unfavourable changes in intra-abdominal adiposity may be more important in the development of CVD than changes in general adiposity. The INTERHEART study also observed a stronger relationship for waist circumference and myocardial infarction than with BMI [36]. This is compatible with the fact that adipose tissue produces and secretes many bioactive molecules such as leptin, adiponectin, angiotensinogen, and inflammatory molecules. These adipokines also interact with other tissues and cells in the body that are linked with CVD [37]. In addition, the more rapid increase in waist circumference, and thus in abdominal fat, in individuals with CVD than in controls, while changes in BMI (a measure of general adiposity) were similar, is consistent with a change in fat distribution in the 10–15 years preceding CVD. If such a change in fat distribution would be accompanied by an overall increase in fat mass, the fact that changes in BMI are similar would likely imply that overall increases in fat mass are accompanied by decreases in muscle mass. This is in line with our secondary analyses, in which we found that, in people with CVD, the decline in eGFR based on cystatin C (-7.3 ml/min/1.73m^2^) was larger than the decline in eGFR based on creatinine (-3.0 ml/min/1.73m^2^) during the 20 years prior to diagnosis (data not shown). Creatinine might remain higher due to a decline in muscle mass, while cystatin C better demonstrates the true decline in kidney function [38, 39].

The presence of unfavourable risk factor levels 15–20 years or more prior to CVD, the fact that CVD event rates increase progressively after the age of 45 years [40–42], and, of course, given that we want to prevent or postpone as many of those cases as possible, underscore the need for CVD prevention in young adulthood. Small elevations in metabolic risk factors and biochemical markers are often not considered clinically relevant at a young age, but our findings indicate that small elevations in most metabolic risk factors and biochemical markers remain the same–they do not disappear–during the 15–20 years preceding CVD. These findings suggests that unfavourable levels are already harmful at a young age, and, they emphasize the importance of maintaining favourable levels of general adiposity, blood pressure, lipids, markers of liver fat, chronic inflammation and kidney function from young adulthood onwards by way of a healthy lifestyle, including a healthy diet and physical activity. Preventing deterioration of systolic blood pressure, abdominal adiposity and kidney function in young adulthood and middle age might be especially warranted since these factors deteriorated more rapidly in people with CVD than controls until diagnosis. In addition, when extrapolating our findings to CVD risk prediction, the relatively small differences in mean levels of metabolic risk factors and biochemical markers between people with CVD and controls underscores the difficulty in differentiating between those who will and those who will not develop CVD long before diagnosis [43]. The similar long-term course of the majority of risk factors in people with CVD and controls also suggests that it is unlikely that multiple measurements of risk factors can improve the performance of CVD prediction models.

Our approach using the modelling of long-term trajectories preceding CVD yields important insights into physiological changes preceding CVD. The strengths of this study are that the same group of trained study personnel objectively measured various metabolic risk factors and biochemical markers in a population-based cohort over a long period. Our study has also certain limitations, in that relatively few participants had measurements of metabolic risk factors and biochemical markers over the full follow-up period due to the limited incidence of CVD after wave 5 (N = 38), which led to relatively large 95% confidence intervals at 20 years or more before diagnosis. Although all types of CVD share common risk factors, the strength of risk factors differs by endpoint. For example, hypertension is more strongly associated with stroke than with coronary heart disease [44, 45]. In sensitivity analyses, we observed only small statistically insignificant differences in levels and trajectories between people with stroke and coronary heart disease. Combining all subtypes of CVD has, therefore, probably led to a small underestimation of the differences in metabolic risk factors and biochemical markers between cases and controls. Furthermore, the non-responders and the excluded participants had slightly more unfavourable levels of the metabolic risk factors and biochemical markers than the study participants. This may have led to an underestimation of the proportion of adults with incident CVD and, as a consequence may have slightly underestimated differences in levels of metabolic risk factors and biochemical markers between people with incident CVD and controls.

## Conclusions

The present study suggests that people with incident CVD have more unfavourable levels, but a similar course, of most metabolic risk factors and biochemical markers compared to controls during the 15–20 years preceding CVD. In contrast, the course of systolic blood pressure, waist circumference and kidney function was more unfavourable in people with CVD than in controls, leading to increasing differences during the 15–20 years preceding diagnosis. These findings seem to indicate that the risk of CVD is already partly determined in young adulthood; thereby stressing the need for primary prevention measures targeted at all risk factors, such as encouraging physical activity and a healthy diet in individuals starting from childhood/young adulthood onwards.

## Supporting Information

S1 Fig Results adjusted for body mass index. **Trajectories of DBP (a), SBP (b), total cholesterol (c), HDL cholesterol (d), triglycerides (e), waist circumference (f), random glucose (g), ALT (h), GGT (i), C-reactive protein (j), uric acid (k), and eGFR (l) of those participants with incident cardiovascular disease (solid black lines) and controls (dashed grey lines) for a hypothetical population of 60 year olds at diagnosis.** Abbreviations: DBP, diastolic blood pressure; SBP, systolic blood pressure; ALT, alanine aminotransferase; GGT, gamma glutamyltransferase; eGFR, estimated glomerular filtration rate. The thin black lines represent the 95% confidence intervals of mean levels of metabolic risk factors and biochemical markers for people with CVD. The thin dashed grey lines represent the 95% confidence intervals of mean levels of metabolic risk factors and biochemical markers for controls. Geometric means are shown for triglycerides, alanine aminotransferase, gamma glutamyltransferase and C-reactive protein. An asterisk (*) indicates a statistically significant difference in trajectory between cases and controls (P<0.10).
(DOCX)

S1 Table Means (standard deviation) and medians (interquartile range) of risk factors and biochemical markers at each wave, separately for those with incident CVD and controls. Abbreviations: ALT, alanine aminotransferase; GGT, gamma glutamyltransferase; eGFR, estimated glomerular filtration rate; NA: not applicable.
(DOCX)

S2 Table Difference in mean level of risk factors and biochemical markers between those with incident CVD and controls 18 years before diagnosis and at diagnosis. Abbreviations: T_-18_, 18 years before diagnosis of cardiovascular disease; ALT, alanine aminotransferase; GGT, gamma glutamyltransferase; eGFR, estimated glomerular filtration rate. *Difference in mean levels between cases and controls tested with t-tests based on the estimated parameters from the random coefficient models.
(DOCX)
