# Supplementary figures and images for: Trajectories of Metabolic Risk Factors and Biochemical Markers prior to the Onset of Cardiovascular Disease – The Doetinchem Cohort Study
Source: PLoS One. 2016 May 20;11(5):e0155978. doi: 10.1371/journal.pone.0155978 (PMC4874669; doi:10.1371/journal.pone.0155978)

**
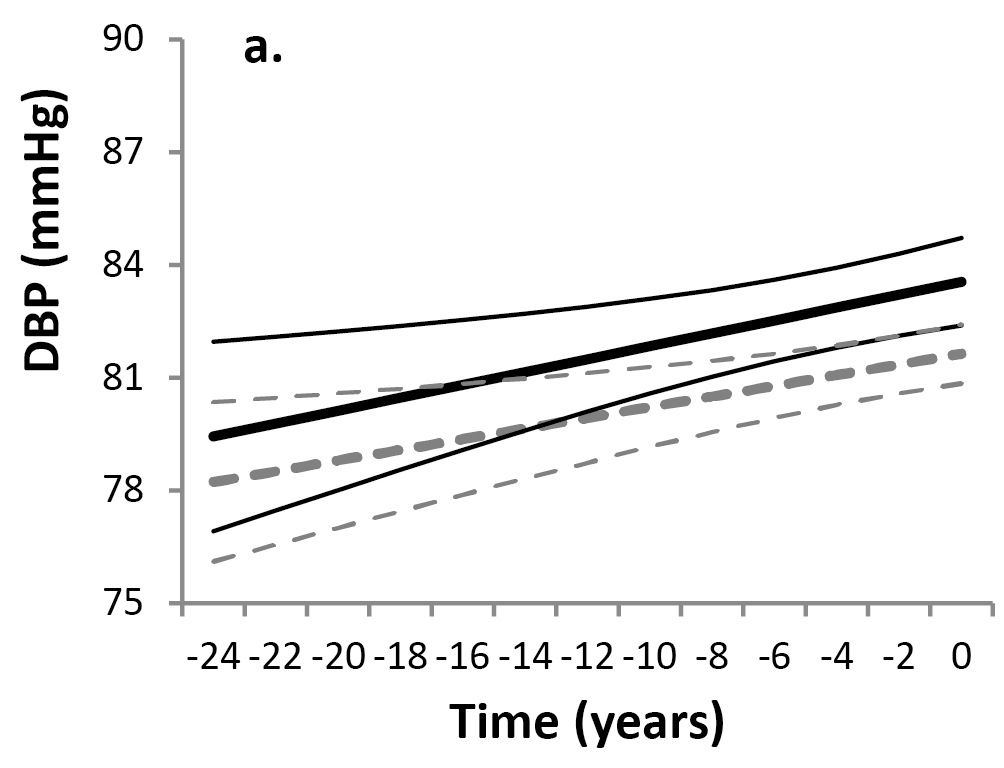

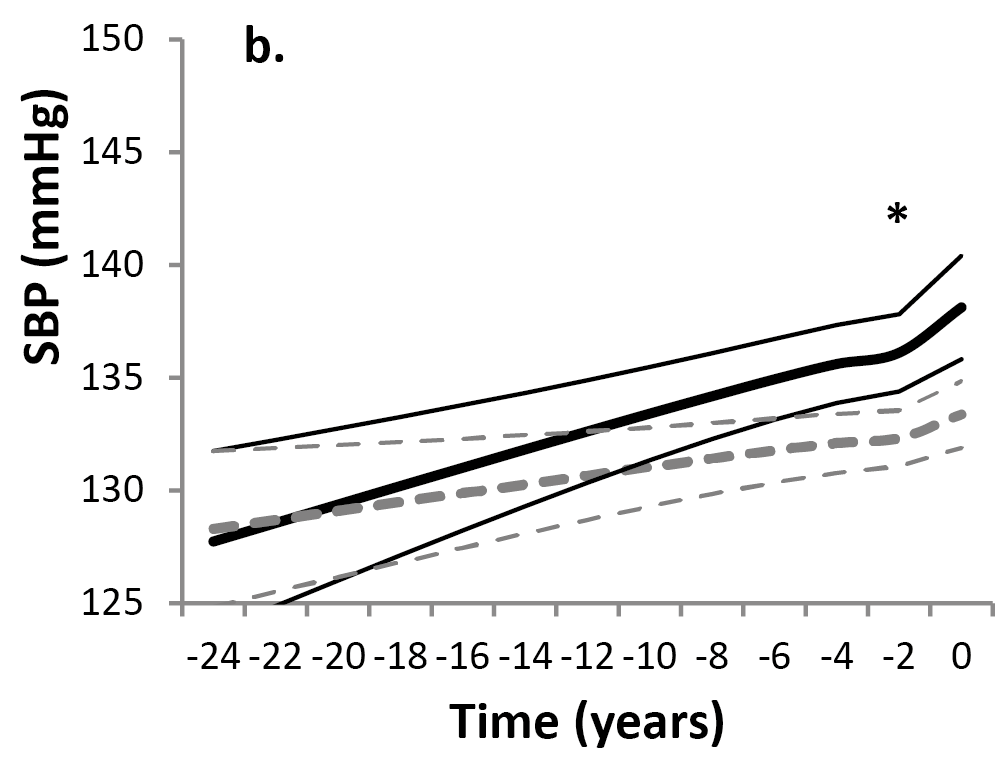

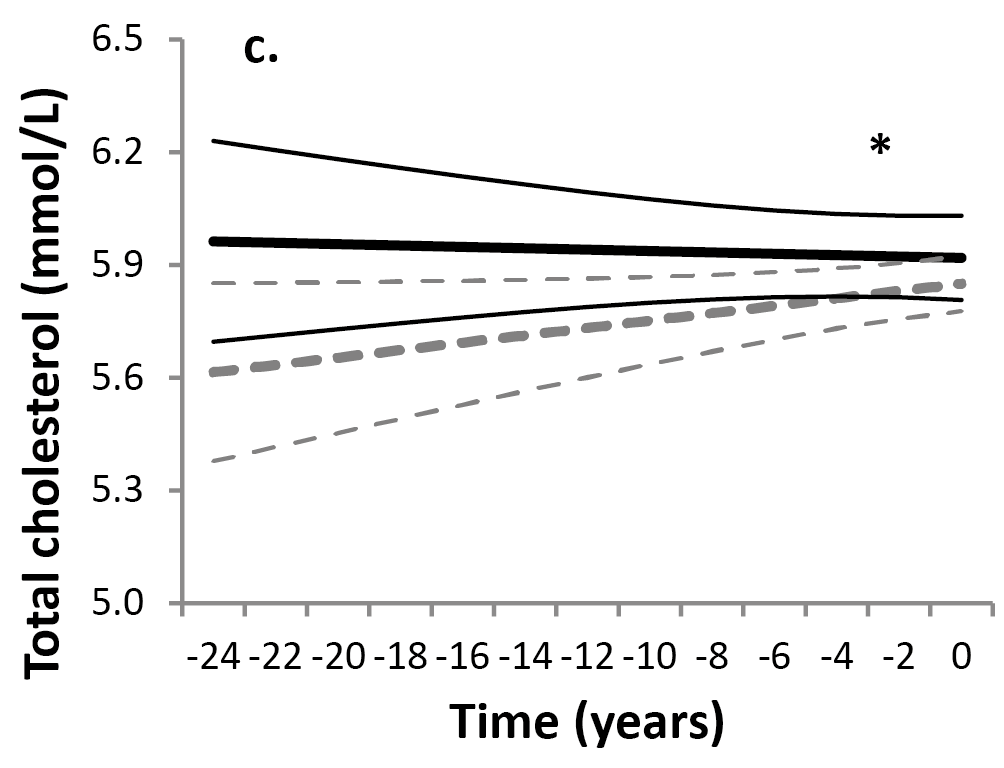

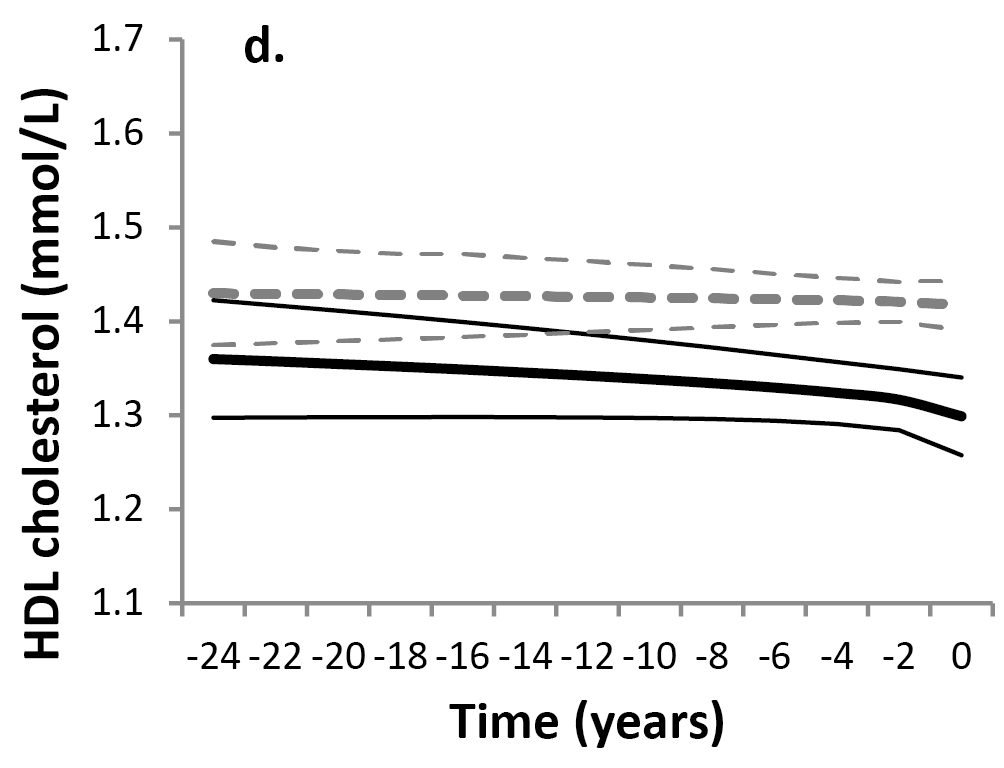

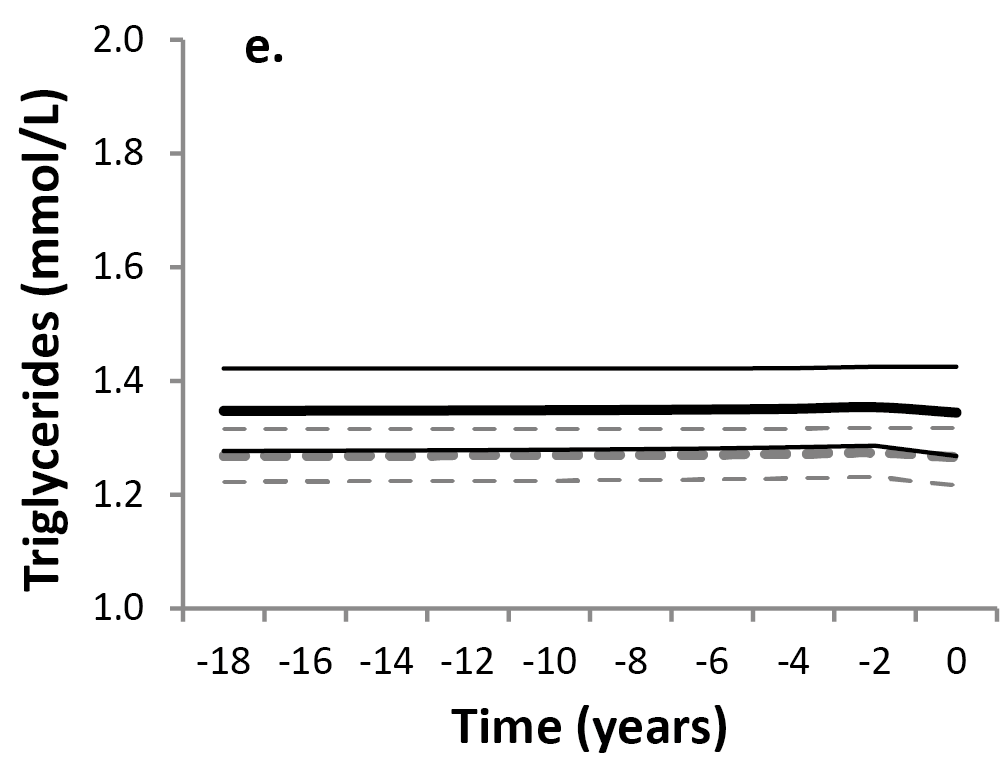

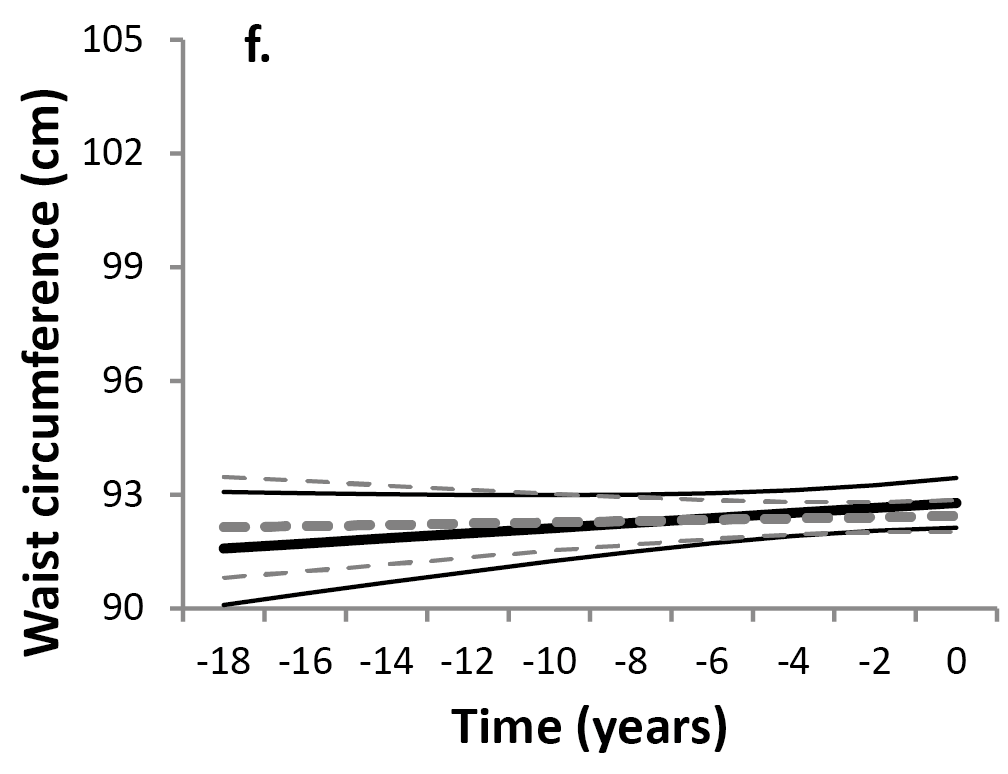

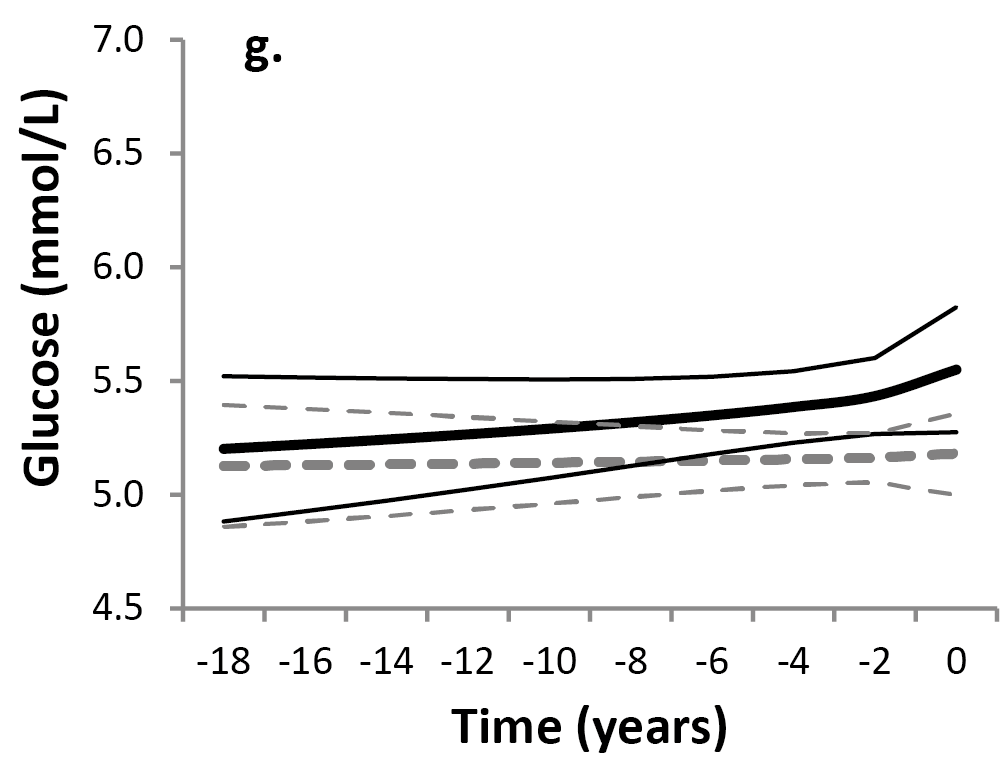

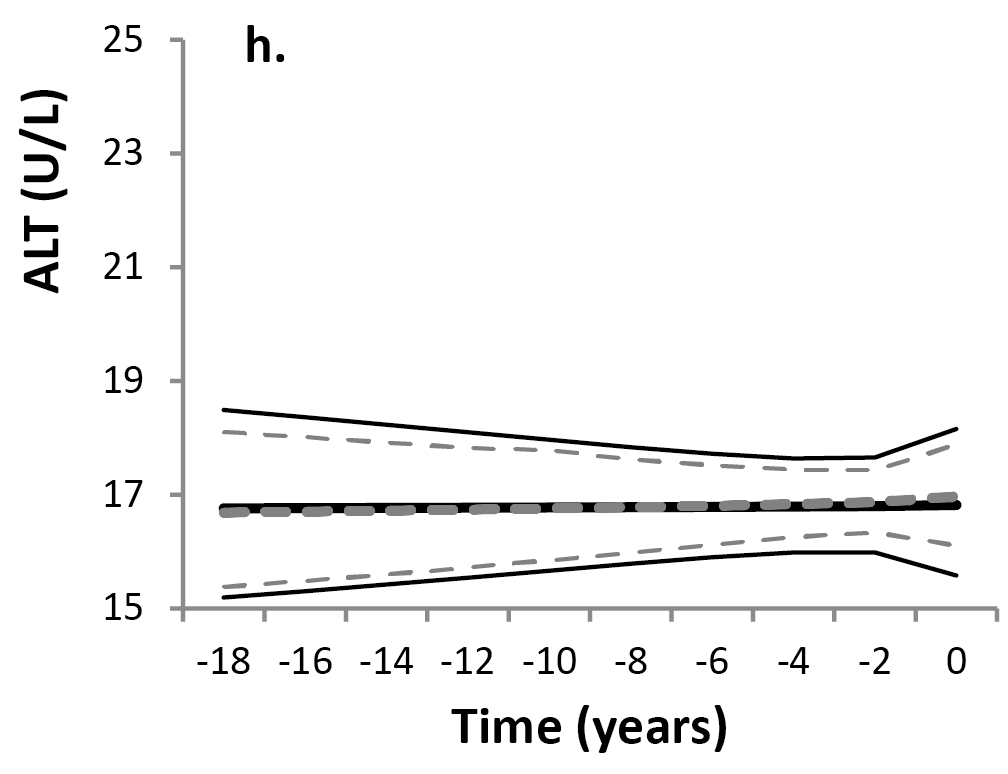
**

**S1 fig.**

**
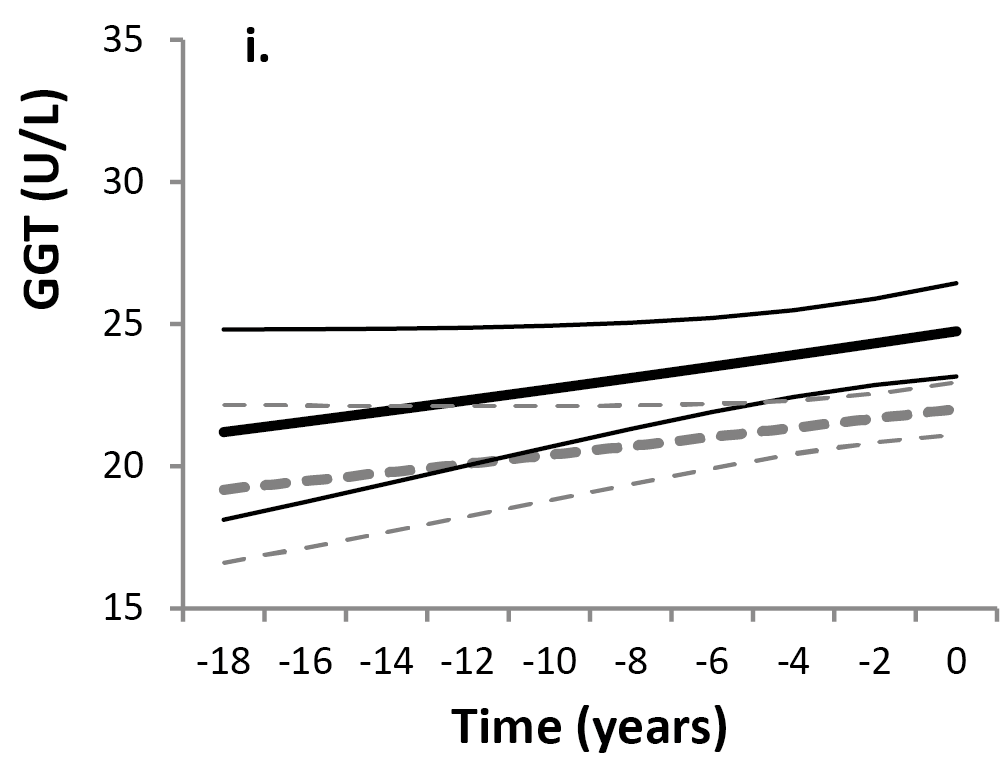

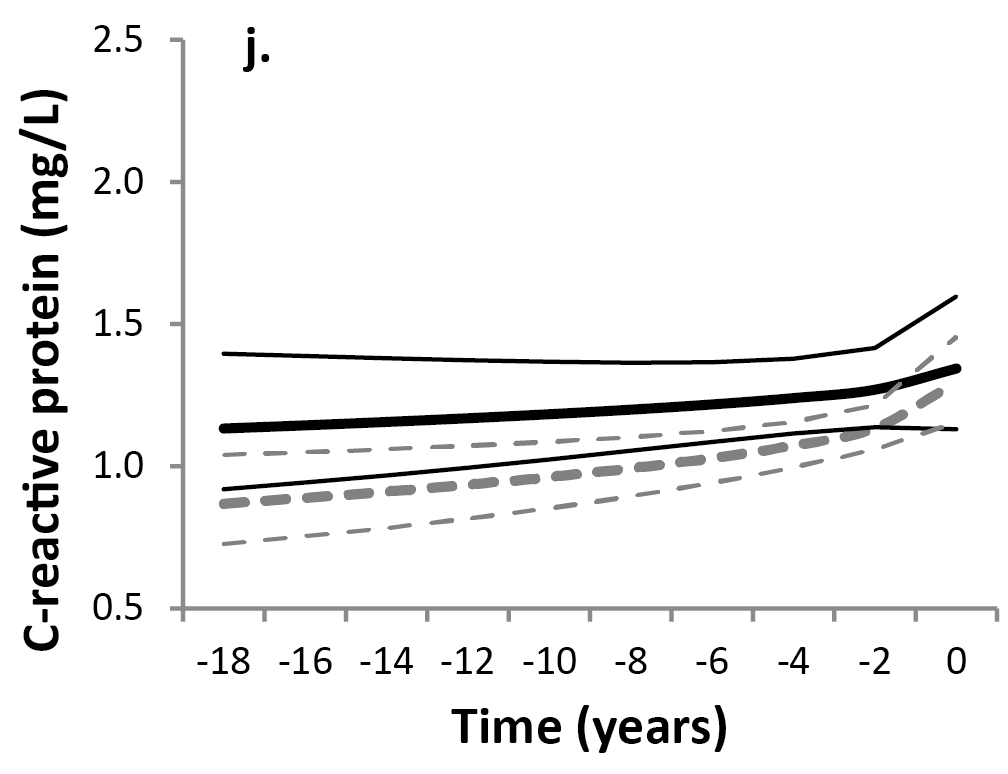

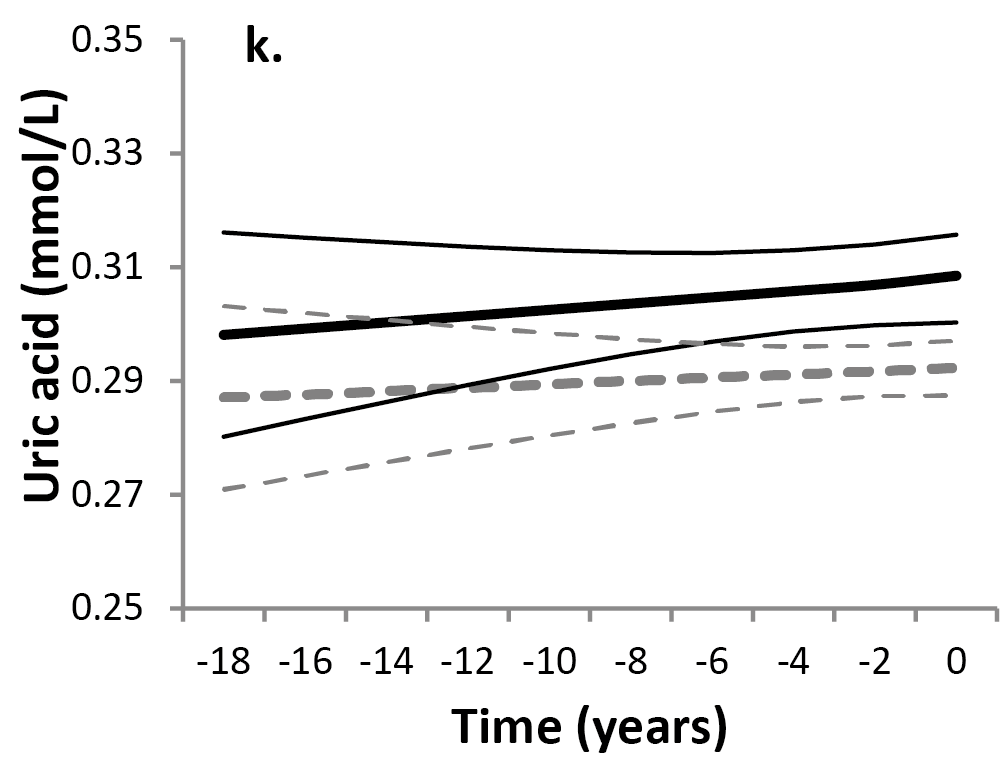

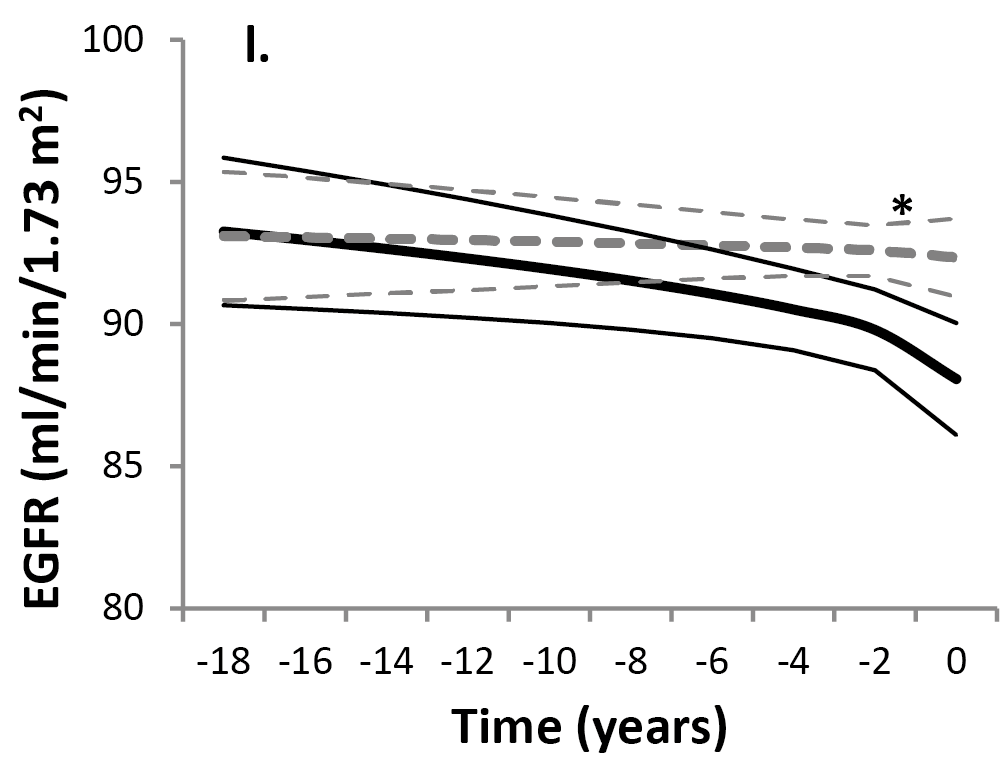
**

**S1 fig** continued.

Supplement: S1 Fig — Trajectories of DBP (a), SBP (b), total cholesterol (c), HDL cholesterol (d), triglycerides (e), waist circumference (f), random glucose (g), ALT (h), GGT (i), C-reactive protein (j), uric acid (k), and eGFR (l) of those participants with incident cardiovascular disease (solid black lines) and controls (dashed grey lines) for a hypothetical population of 60 year olds at diagnosis. Abbreviations: DBP, diastolic blood pressure; SBP, systolic blood pressure; ALT, alanine aminotransferase; GGT, gamma glutamyltransferase; eGFR, estimated glomerular filtration rate. The thin black lines represent the 95% confidence intervals of mean levels of metabolic risk factors and biochemical markers for people with CVD. The thin dashed grey lines represent the 95% confidence intervals of mean levels of metabolic risk factors and biochemical markers for controls. Geometric means are shown for triglycerides, alanine aminotransferase, gamma glutamyltransferase and C-reactive protein. An asterisk (*) indicates a statistically significant difference in trajectory between cases and controls (P<0.10). (DOCX) [file pone.0155978.s001.docx]
