# Supplementary material for: Trajectories of Metabolic Risk Factors and Biochemical Markers prior to the Onset of Cardiovascular Disease – The Doetinchem Cohort Study
Source: PLoS One. 2016 May 20;11(5):e0155978. doi: 10.1371/journal.pone.0155978 (PMC4874669; doi:10.1371/journal.pone.0155978)
Supplement: S1 Table — Abbreviations: ALT, alanine aminotransferase; GGT, gamma glutamyltransferase; eGFR, estimated glomerular filtration rate; NA: not applicable. (DOCX) [file pone.0155978.s002.docx]

**S1 Table. Mean (standard deviation) and median (interquartile range) of metabolic risk factors and biochemical markers at each wave, separately for those with incident CVD and controls.**

|  | Wave 1 | | Wave 2 | | Wave 3 | | Wave 4 | | Wave 5 | |
| --- | --- | --- | --- | --- | --- | --- | --- | --- | --- | --- |
|  | Cases | Controls | Cases | Controls | Cases | Controls | Cases | Controls | Cases | Controls |
| Body mass index (kg/m^2^) | 27.3 (4.0) | 26.5 (3.7) | 26.7 (3.8) | 26.0 (3.6) | 26.2 (3.5) | 25.5 (3.4) | 25.4 (3.4) | 24.9 (3.3) | 26.8 (3.4) | 24.7 (2.8) |
| Diastolic blood pressure (mmHg) | 85 (12) | 83 (10) | 84 (11) | 81 (11) | 83 (11) | 80 (11) | 80 (10) | 78 (10) | 79 (10) | 78 (10) |
| Systolic blood pressure (mmHg) | 138 (19) | 133 (18) | 133 (17) | 129 (17) | 129 (17) | 126 (16) | 125 (15) | 122 (15) | 123 (13) | 122 (15) |
| Total cholesterol (mmol/L) | 5.8 (1.1) | 5.7 (1.0) | 6.0 (1.2) | 5.8 (1.0) | 5.9 (1.1) | 5.7 (1.0) | 5.8 (1.2) | 5.6 (1.0) | 6.1 (1.4) | 5.7 (1.0) |
| HDL cholesterol (mmol/L) | 1.25 (0.38) | 1.35 (0.37) | 1.20 (0.35) | 1.30 (0.34) | 1.22 (0.35) | 1.30 (0.34) | 1.20 (0.29) | 1.23 (0.30) | 1.07 (0.28) | 1.23 (0.28) |
| Triglycerides (mmol/L) | NA | NA | 1.6 (1.1-2.1) | 1.4 (1.0-2.0) | 1.5 (1.1-2.1) | 1.4 (1.0-1.9) | 1.3 (1.0-1.9) | 1.3 (1.0-1.8) | 1.7 (1.0-2.7) | 1.4 (1.0-2.0) |
| Glucose (mmol/L) | NA | NA | 5.8 (1.9) | 5.4 (1.5) | 5.7 (1.9) | 5.4 (1.3) | 5.6 (1.8) | 5.3 (1.1) | 5.6 (1.6) | 5.5 (1.3) |
| Waist circumference (cm) | NA | NA | 99 (12) | 97 (11) | 96 (12) | 95 (12) | 94 (10) | 93 (11) | 96 (11) | 93 (9) |
| ALT (U/L) | NA | NA | 18 (14-23) | 17 (13-22) | 19 (14-25) | 17 (13-23) | 17 (15-24) | 18 (14-25) | 19 (11-27) | 22 (16-27) |
| GGT (U/L) | NA | NA | 26 (19-40) | 23 (16-35) | 26 (18-41) | 23 (16-34) | 23 (16-35) | 21 (15.33) | 26 (20-42) | 23 (17-35) |
| C-reactive protein (mg/L) | NA | NA | 1.5 (0.7-3.3) | 1.3 (0.7-2.5) | 1.5 (0.7-2.9) | 1.1 (0.6-2.1) | 1.3 (0.6-2.8) | 1.0 (0.5-2.0) | 1.8 (0.8-3.2) | 0.9 (0.5-1.5) |
| Uric acid (mmol/L) | NA | NA | 0.33 (0.08) | 0.31 (0.07) | 0.32 (0.07) | 0.30 (0.07) | 0.31 (0.08) | 0.29 (0.07) | 0.32 (0.08) | 0.31 (0.07) |
| eGFR (ml/min/1.73 m^2^) | NA | NA | 86 (15) | 88 (13) | 91 (14) | 91 (13) | 94 (13) | 94 (13) | 98 (11) | 96 (13) |

Abbreviations: ALT, alanine aminotransferase; GGT, gamma glutamyltransferase; eGFR, estimated glomerular filtration rate; NA: not applicable.
