# Supplementary material for: Trajectories of Metabolic Risk Factors and Biochemical Markers prior to the Onset of Cardiovascular Disease – The Doetinchem Cohort Study
Source: PLoS One. 2016 May 20;11(5):e0155978. doi: 10.1371/journal.pone.0155978 (PMC4874669; doi:10.1371/journal.pone.0155978)
Supplement: S2 Table — Abbreviations: T-18, 18 years before diagnosis of cardiovascular disease; ALT, alanine aminotransferase; GGT, gamma glutamyltransferase; eGFR, estimated glomerular filtration rate. *Difference in mean levels between cases and controls tested with t-tests based on the estimated parameters from the random coefficient models. (DOCX) [file pone.0155978.s003.docx]

**S2 Table. Difference in mean level of risk factors and biochemical markers between those with incident CVD and controls 18 years before diagnosis and at diagnosis.**

|  | T_-18_ | Diagnosis |
| --- | --- | --- |
|  | β (95%CI) * | β (95%CI) |
| Body mass index (kg/m^2^) | 0.1 (-0.6;0.8) | 0.7 (0.4;1.1) |
| Diastolic blood pressure (mmHg) | 1.2 (-0.7;3.1) | 2.4 (1.2;3.6) |
| Systolic blood pressure (mmHg) | 2.8 (-0.2;5.9) | 5.5 (3.1;7.9) |
| Total cholesterol (mmol/L) | 0.1 (-0.1;0.3) | 0.1 (-0.02;0.2) |
| HDL cholesterol (mmol/L) | -0.07 (-0.13;-0.0003) | -0.12 (-0.16;-0.07) |
| Triglycerides (log mmol/L) | 0.09 (0.03;0.15) | 0.09 (0.03;0.15) |
| Glucose (mmol/L) | 0.5 (0.2;0.8) | 0.3 (0.04;0.6) |
| Waist circumference (cm) | 2 (-1;5) | 2 (1;4) |
| ALT (log U/L) | 0.03 (-0.10;0.16) | 0.02 (-0.06;0.10) |
| GGT (log U/L) | 0.06 (-0.11;0.23) | 0.16 (0.09;0.23) |
| C-reactive protein (log mg/L) | 0.2 (-0.1;0.5) | 0.1 (-0.1;0.3) |
| Uric acid (mmol/L) | 0.002 (-0.018;0.022) | 0.020 (0.011;0.028) |
| eGFR (ml/min/1.73 m^2^) | 1.0 (-2.4;4.4) | -4.6 (-6.6;-2.7) |

Abbreviations: T_-18_, 18 years before diagnosis of cardiovascular disease; 95%CI, 95% confidence interval; ALT, alanine aminotransferase; GGT, gamma glutamyltransferase; eGFR, estimated glomerular filtration rate.

*Difference in mean levels between cases and controls tested with t-tests based on the estimated parameters from the random coefficient models.
